# Effects of continuous and slow tracheal tube cuff deflation on cough reflex during extubation in noncardiac surgery patients: a randomised clinical trial

**DOI:** 10.1186/s12871-023-02003-5

**Published:** 2023-02-07

**Authors:** Xuan Wang, Guangli Zhu, Jing Tan, Xinyu Cao, Manlin Duan

**Affiliations:** 1 Department of Anesthesiology, Benq medical center & Jinling Hospital, Jinling School of Clinical Medicine, Nanjing Medical University, No. 71 Hexi Avenue, Jianye District, Jiangsu Province 210019 Nanjing, People’s Republic of China; 2 Jiangsu Provincial Key Laboratory of Anesthesiology, Xuzhou Medical University, Xuzhou, Jiangsu Province People’s Republic of China; 3 Department of Anesthesiology, Jiangsu Cancer Hospital & Jiangsu Institute of Cancer Research & The Affiliated Cancer Hospital of Nanjing Medical University, Nanjing, Jiangsu Province People’s Republic of China

**Keywords:** Cough reflex, Cuff pressure gauge, Deflation, Postoperative airway complications, Tracheal extubation

## Abstract

**Background:**

The incidence of cough reflex during extubation is 76%. Cough reflex causes severe hemodynamic fluctuations and airway complications. This prospective trial investigated the potential effects of tracheal tube cuff deflation on cough reflex during extubation.

**Methods:**

One hundred and twenty-six patients scheduled for operations within 3 h under general anaesthesia with orotracheal intubation were randomly assigned to one of three groups: control (C), experimental (E) or syringe (S) groups. Patients in group C underwent tracheal tube cuff deflation using a 10-ml syringe in 1 s, patients in group E underwent tracheal tube cuff deflation continuously and slowly in 5 s using a cuff pressure gauge until the pressure was zero and patients in group S underwent tracheal tube cuff deflation using a 10-ml syringe at a speed of 1 ml s^−1^. The incidence and severity of cough reflexs during extubation and the incidence of postoperative airway complications within 48 h were assessed.

**Results:**

Compared with group C (60.0%), the incidence of cough reflex in group E was 9.8% (*p* < 0.001) and in group S was 12.5% (*p* < 0.001). The severity of cough reflex was graded as 2 (1–2) in group C, 1 (1–1) in group E and 1 (1–1) in group S (*p* < 0.001 for group comparisons). The incidence of hoarseness in group C was 0.0%, in group E was 19.5% and in group S was 5.0% (*p* < 0.05 for all groups, *p* = 0.009 between group C and E).

**Conclusions:**

Compared with deflating a trachal tube cuff with a 10-ml syringe in 1 s, the use of a 10-ml syringe at a speed of 1 ml s^−1^ or a cuff pressure guage within 5 s can both reduce the incidence of cough reflex, but deflating with a cuff pressure guage can increase the incidence of postoperative hoarseness.

**Trial registration:**

Chinese Clinical Trial Registry, identifier: ChiCTR2100054089, Date: 08/12/2021.

## Background

Cough reflex from general anaesthesia in the presence of an endotracheal tube has an incidence of 15–94% [1–3], and during extubation was as high as 76% [4]. One of the factors is the endotracheal tube cuff inflation, it can produce significant mechanical irritation of the patient’s larynx [5] and during the recovery period of general anaesthesia, the depth of anaesthesia is gradually reduced, and the patient's muscle strength and pharyngeal reflex are gradually restored.

Cough reflex is often accompanied with violent hemodynamic fluctuations, increased intracranial pressure and increased intraocular pressure, which can lead to cardiovascular and cerebrovascular complications. Cough reflex can cause postoperative airway complications, such as sore throat, hoarseness, airway spasms, and laryngeal mucosal haemorrhage [6].

Various strategies aimed at reducing cough reflex caused by endotracheal tube cuff have been studied, such as applying local anaesthetics to the endotracheal tube cuff, inhaling steroid preoperatively, opioid or lidocaine administration during emergence and extubation, or extubation under deep anesthesia [7–9]. No trial on cough reflex caused by sudden pressure reduction of the endotracheal tube cuff during extubation has been conducted. Based on personal observations, we hypothesized that continuous and slow tracheal tube cuff deflation can effectively suppress the cough reflex.

We conducted this study to investigate whether continuously and slowly deflating the tracheal tube cuff was associated with a lower incidence and severity of cough reflex and airway complications compared with rapid deflation.

## Methods

### Ethics

The protocol was approved by the Research Ethics Committee of Jinling Hospital, Jinling School of Clinical Medicine, Nanjing Medical University (Ethical Application Reference: 2022DZKY-024–01 Nanjing, China) on 18 March 2022. All methods were performed in accordance with relevant guidelines and regulations with CONSORT recommendations [10]. The study was registered in the Chinese Clinical Trial Registry (Identifier: ChiCTR2100054089, Date: 08/12/2021). Before participation, all the patients and / or their legal guardians provided written informed consent.

### Study design and population

In this trial, 126 patients with ASA physical status I or II aged 18–65 years who were prearranged for operations within 3 h under general anaesthesia with orotracheal intubation were included between March 2022 and June 2022. The exclusion criteria were incapacity to provide informed consent; body mass index < 19 kg m^−2^ or > 30 kg m^−2^; Mallampati classification III or IV; pre-existing sore throat, hoarseness, cough or laryngeal mucosa haemorrhage; intubation more than once when induction; bucking during surgery; delayed emergence (inability to regain an adequate level of consciousness, unresponsive or deeply sedated over 60 min from the last administration of the anesthetic agents [11, 12]); reintubation after extubation within 48 h; asthma, chronic obstructive pulmonary disease or smoking; psychiatric disorders; and brain surgery, laparoscopic surgery, ear-nose-throat surgery and nasotracheal intubation.

### Randomisation

The online computer system ‘OPEN-randomize’ was used to randomly assign patients to the groups, and it generated the randomization sequence to ensure equal distribution [13]. No restrictions applied for random selection, and the numbers for allocation were packaged in opaque envelopes, which could be observed by the anesthesiologist, who has more than 5 years of experience and conducted intubation and extubation. Patients, two outcome evaluators, data information analysts were blinded to the trial intervention. As it was difficult to fully blind the evaluator if he assessed the cough reflex and the hemodynamic changs at the same time, so we recorded video to assess the incidence and severity of cough reflex by an independent evaluator and other outcomes were assessed by another evaluator.

Patients in the control (C) group (*n* = 42) underwent endotracheal tube cuff deflation using a 10-ml syringe in 1 s, those in the experimental (E) group (*n* = 42) underwent endotracheal tube cuff deflation by same amount of pressure reduction each second until the pressure was zero continuously and slowly with a cuff pressure gauge (Ambu*R, Germany) in 5 s, and the pressure guage still attached when removing the tube because this would allow the cuff to further deflate when passing the vocal cords. Patients in the syringe (S) group (*n* = 42) underwent endotracheal tube cuff deflation using a 10-ml syringe at a speed of 1 ml s^−1^.

### Anaesthesia protocol and extubation

All the patients received standardized monitoring procedures after entering the operation room: electrocardiography, the saturation of haemoglobin with oxygen, non-invasive blood pressure and bispectral index (BIS). The patients were induced with sufentanil 0.4 μg kg^−1^, propofol 2.0 mg kg^−1^ and cisatracurium 0.2 mg kg^−1^. An endotracheal tube reinforced with steel and a tapered cuff (cuffed, Hisern medical, Zhejiang, China) was selected. The tube size was determined by the inner diameter. We selected 7.0 or 7.5 mm for females and 7.5 or 8.0 mm for males, and an anaesthesiologist with > 5 years’ experience intubated the patients within 30 s by a glidescope. The endotracheal tube cuff was inflated with 5–8 ml room-temperature air, and the cuff pressure was strictly maintained at 25–30 cmH_2_O during anaesthesia. Anaesthesia was maintained using remifentanil 0.5 µg kg^−1^ min ^−1^, propofol 4–8 mg kg^−1^ h^−1^ and cisatracurium 0.05 mg kg^−1^ every 30 min. Intraoperative mechanical ventilation was determined according to the institution protocol (volume-controlled ventilation; tidal volume 6–8 mL kg^−1^ of ideal body weight, initial respiratory rate of 10–12 breaths min^−1^_,_ *I*:*E* ratio of 1:2, PEEP was 0 cm H_2_O, peak airway pressure was below 25 mmHg). The BIS value was maintained at 40–60, end-tidal CO_2_ (EtCO_2_) was maintained at 35–45 mmHg and blood pressure fluctuation was within ± 20%. Cisatracurium was no longer added 30 min before the end of surgery. Before subcuticular closure, the administration of propofol and remifentanil were stopped and 0.2 μg kg^−1^ sufentanil was injected intravenously. Postoperatively, all patients were transported to the post-anaesthesia care unit (PACU).

During recovery from general anaesthesia in PACU, sputum around the airway and endotracheal tube cuff could be suctioned when the patients were still under deep anaesthesia. No medications were given except neostigmine and atropine to antagonize residual neuromuscular block before extubation. Once the patients conformed to the indications of extubation (train of four ratio (TOF) was ≥ 0.9, EtCO_2_ was < 45 mmHg on spontaneous respiration and could follow voice commands), endotracheal tube cuff deflation was performed by the anesthesiologist who intubated the patient and then the tracheal tube was slowly and gently extubated.

All the patients received patient-controlled intravenous analgesia postoperatively: sufentanil 50 μg; dexmedetomidine 200 μg and ondansetron 8 mg diluted to 100 ml with 0.9% sodium chloride. The dose was controlled at 1.5 ml at all times, the rate was 2 ml h ^−1^, and was locked for 15 min.

### Outcome measures

The primary goal was to determine the incidence and severity of cough reflex when extubation and immediate post-extubation within first second (mentioned as extubation immediately). The incidence of postoperative airway complications, such as apnoea, hypoxaemia, airway spasm within 30 min and sore throat, hoarseness, laryngeal mucosa haemorrhage within 48 h were the secondary outcomes. Furthermore, mean artery pressure (MAP) and heart rate (HR) were measured at various time points after entering the operating room (baseline), extubation immediately and 1, 10 and 30 min later. Operation duration, emergence duration (from the end of surgery to extubation) and anaesthesia duration (from anaesthesia induction to extubation) were also recorded. Cough was graded on 0–3 scales as follows: 0 = no cough, 1 = single cough, 2 = more than one episode of unsustained cough, 3 = severe sustained bouts of cough [14]. Unsustained cough was defined as less than 5 s between two coughs and cough reflex was defined as cough score > 0.

### Sample size and statistical analysis

According to published data, the incidence of cough reflex during extubation can reach 76% [4]. If a 50% reduction in cough reflex incidence was determined to be clinically significant, with an effect size of 0.36, degrees of freedom of 2, a power of 90%, and an error of 0.05, Chi-Square test by the Power Analysis and Sample Size Software (version 15.0; NCSS, LLC, USA) calculated that 99 patients were required totally. Given a 20% dropout rate, 126 patients were included, each group was 42 patients. According to the normality of the distribution, continuous study variables were summarized as mean (standard deviation) or median (25–75^th^ percentiles). Frequencies were used to summarize categorical variables (percentage). The Shapiro–Wilk test was used to determine normality. For normally distributed continuous variables, group differences were tested using one-way analysis of variance (ANOVA). The Levene test for homogeneity of variance was performed, and *p* > 0.1 was considered homogeneous of variance. If the ANOVA result was statistically significant, least significant difference (LSD) was performed for pairwise comparison when the variance between the groups was homogeneous; otherwise, Dunnett T3 was used. For non-normally distributed continuous variables, the Kruskal–Wallis test was used, and if the result was statistically significant, the Mann–Whitney test was used for pairwise comparison. Categorical variables were compared using the chi-square test. SPSS version 19.0 was used to conduct the statistical analyses (SPSS Inc., Chicago, IL, USA). The reported p-values were Bonferroni-corrected. *p* < 0.05 was regarded as statistically significant.

## Results

**Fig. 1:**
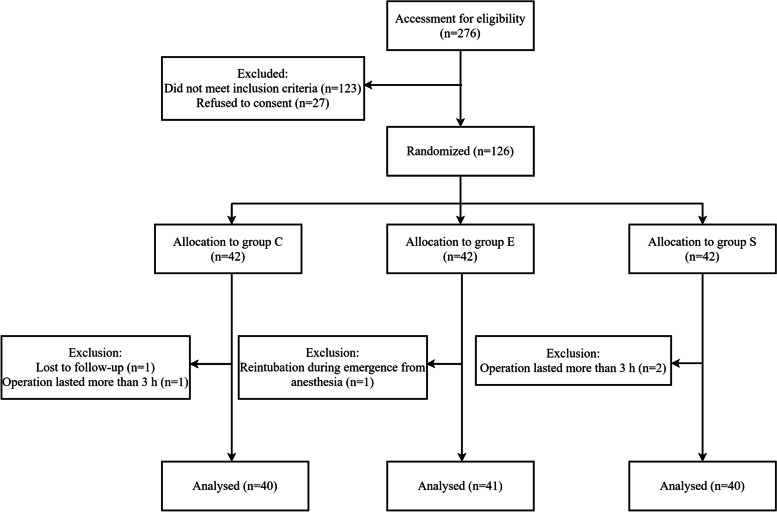
Consort flow chart that outlines patients’ assignment and different methods of extubation. Patients were randomly assigned into three groups (groups C, E and S) to receive different methods of tracheal tube cuff deflation, following a computer-generated randomization code

This study enrolled 126 of 276 eligible patients, and 5 were excluded from the analysis: 3 had an operation that lasted more than 3 h, 1 was lost to follow-up, 1 had reintubation during emergence from anesthesia (Fig. 1).

The characteristics of the patients and the operations were comparable across the groups (Table 1). There were no differences in age, sex, BMI, ASA classification, Mallampati classification or sort of surgeries among the three groups. There were no significant differences among the groups in terms of the patients' tracheal tube size, the cuff inflation volumes when intubation, operation duration, anaesthesia duration and emergence duration (Table 1).

**Table 1:** Patients and operation characteristics

	Group C (*n* = 40)	Group E (*n* = 41)	Group S (*n* = 40)
Age (yr)	48.0 (42.3–54.0)	47.0 (33.0–55.5)	47.5 (36.3–55.8)
Female, n(%)	17 (42.5)	15 (36.7)	21 (52.5)
BMI (Kg m^−2^)	23.4 (21.6–25.7)	23.4 (22.1–26.3)	22.2 (22.0–24.4)
ASA classification, n(%)			
I	2 (5.0)	5 (12.2)	4 (10.0)
II	38 (95.0)	36 (87.8)	36 (90.0)
Mallampati classification, n(%)			
I	12 (30.0)	10 (24.4)	13 (32.5)
II	28 (70.0)	31 (75.6)	27 (67.5)
Operation duration (min)	116.6 ± 38.5	110.1 ± 38.9	111.6 ± 35.7
Emergence duration (min)	43.2 ± 9.0	46.5 ± 7.8	45.9 ± 7.0
Anaesthesia duration (min)	163.9 ± 47.9	148.4 ± 44.1	149.6 ± 41.1
Cuff volume (ml)	6.0 (5.7–7.0)	6.0 (5.0–6.5)	6.0 (5.0–6.8)
Tube size, n(%)			
7.0	14 (35.0)	11 (26.8)	16 (40.0)
7.5	26 (65.0)	29 (70.7)	23 (57.5)
8.0	0 (0.0)	1 (2.5)	1 (2.5)
Sort of Surgeries, n(%)			
Lower extremity	19 (47.5)	22 (53.7)	18 (45.0)
Urological	8 (20.0)	7 (17.0)	11 (27.5)
abdominal	13 (32.5)	12 (29.3)	11 (27.5)

Abbreviations: *BMI* Body Mass Index, *ASA* American Society of Anesthesiologists. *SD* standard deviation. The values are expressed as mean ± SD, median (25-75^th^ percentiles), or number of patients (percentage). Age is mean (range)

*P* < 0.05 is considered statistic significant

**Fig. 2:**
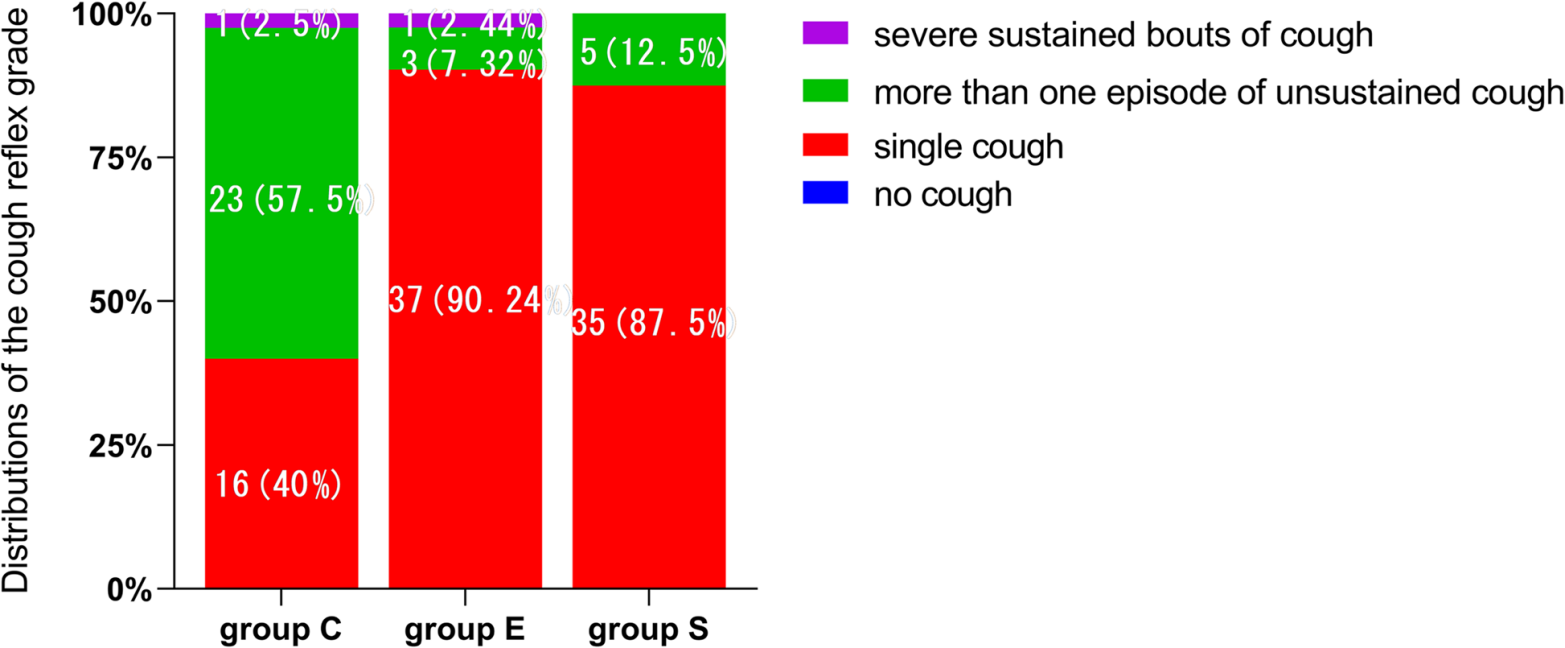
Distrubtion of cough reflex grade in 3 groups. The severity of cough reflex was graded as 2 (1–2) in group C, 1 (1–1) in group E, and 1 (1–1) in group S (*p* < 0.001 for group comparisons). It was graded on 0–3 scales. Grade of cough: 0 = no cough, 1 = single cough, 2 = more than one episode of unsustained cough, 3 = severe sustained bouts of cough

The incidence of cough reflex was 60.0% in group C, 9.8% in group E and 12.5% in group S ( *p* < 0.001 for all groups, group C was higher than group E and S, *p* < 0.001 for both comparisons, Table 2). The severity of cough reflex was graded as 2 (1–2) in group C, 1 (1–1) in group E, and 1 (1–1) in group S (*p* < 0.001 for group comparisons, Fig. 2).

**Table 2:** Incidence and severity of cough reflex and postoperative airway complications

	Group C (*n* = 40)	Group E (*n* = 41)	Group S (*n* = 40)	*P* values
Primary outcome				
Incidence of cough reflex, n (%)	24 (60.0)	4 (9.8)^*^	5 (12.5)^*^	< 0.001
Grade of cough reflex, (score)	2 (1–2)	1 (1–1)^*^	1 (1–1)^*^	< 0.001
Secondary outcome				
Incidence of complications, n (%)	6 (15.0)	13 (31.7)	5 (12.5)	0.062
Hoarseness, n(%)	0 (0.0)	8 (19.5)^#^	2 (5.0)	0.04
Sore throat, n(%)	3 (7.5)	4 (9.8)	3 (7.5)	0.913
Others, n(%)	3 (7.5)	1 (2.4)	0 (0.0)	0.160

Abbreviations: *SD* standard deviation. The values are expressed as mean ± SD, median (25-75^th^percentiles), or number of patients (percentage)

*P *< 0.05 is considered statistic significant

^*^P < 0.001 vs group C

^#^*P* < 0.05 vs group C

The incidence of postoperative airway complications in group C was 15.0%, in group E was 31.7% and in group S was 12.5% (*p* = 0.062, Table 2). To further analyse, the postoperative airway complications were mainly hoarseness and sore throat. The incidence of hoarseness in group C was 0.0%, in group E was 19.5% and in group S was 5.0% (*p* < 0.05 for all groups, *p* = 0.009 between group C and E, Table 2). The incidence of sore throat in group C was 7.5%, in group E was 9.8% and in group S was 7.5% (*p* = 0.913, Table 2). The incidence of other postoperative airway complications including apnoea, hypoxaemia, airway spasm within 30 min and laryngeal mucosa haemorrhage within 48 h in group C was 7.5%, in group E was 2.4% and in group S was 0.0% (*p* = 0.160, Table 2).

**Fig. 3:**
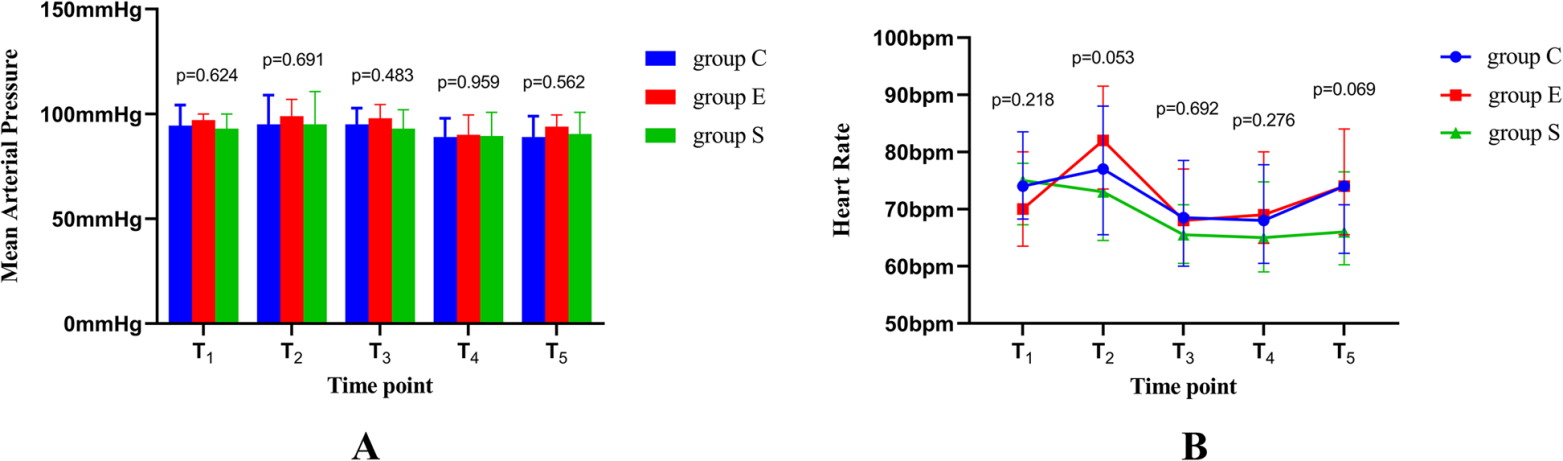
**A**, MAP at various time points; **B**, HR at various time points. There was no difference in MAP and HR among baseline, extubation immediately and 1, 10, 30 min later. T_1_, baseline. T_2_, extubation immediately. T_3_, 1 minute after extubation. T_4_, 10 minutes after extubation. T_5_, 30 minutes after extubation. MAP, mean arterial pressure. HR, heart rate

There was no difference in MAP or HR among baseline, extubation immediately and 1, 10, 30 min later (Fig. 3).

## Discussion

Compared with deflating a tracheal tube cuff with a 10-ml syringe in 1 s, deflating continuously and slowly until the pressure reduce to zero via a cuff pressure gauge in 5 s or using a 10-ml syringe at a speed of 1 ml s^−1^ can both reduce the incidence and severity of cough reflex. However, the use of a cuff pressure guage to deflate the cuff increased the incidence of postoperative hoarseness.

In this trial, we adopted a cuff pressure gauge for deflation to modulate the speed of deflation according to cuff pressure objectively. We designed a group to deflate with a 10 ml syringe at a speed of 1 ml s^−1^, which we believe was similar to the deflating speed via a cuff pressure gauge for convenience in clinical practice. The incidence of cough reflex in group C was 60.0%, which was confirmed in 15–94%,^3^ however, it was lower than 76%, we estimated it was because of total intravenous anesthesia rather than isoflurane anesthesia and the sufentanil injection before subcuticular closure. Numerous studies have reported intravenous medications such as opioids, dexmedetomidine [3, 15, 16] or corticosteroids, water-soluble lubrication, and local anaesthetics such as lidocaine applied to the surface of the cuff [17–19] can suppress cough reflex at the time of extubation. However, medication preventions have limitations. For instance, opioids can cause opioid induced respiratory depression, the effects of local anaesthetics depend on the type and concentration used. In addition, the protective cough reflexes above the tube cuff and of the vocal cords should remain intact. Up to now, no trials evaluated whether deflating the tracheal tube cuff continuously and slowly can reduce the incidence of cough reflex, which represents relieving the pressure on the trachea continuously and slowly.

Tracheal tube cuff is in closest contact with the tracheal mucosa, so most stimulation come from the cuff [20]. Extubation can stimulate reflex responses via irritanting or stretching trachea and larynx [21]. And rapidly acting receptors are found throughout the trachea and are primarily superficial. They are thought to be the irritant receptors involved in the cough reflex [20]. The underlying mechanisms of cough suppression with slow and continuous endotracheal tube cuff deflation are not clearly understood. We estimated that slow and continuous deflation lead to a mild release of pressure on trachea, which may reduce stimulation intensity on rapidly acting receptors. Airway sensory C fibers is high sensitive to chemical stimulation and moderate sensitive to physical stimulation [22]. Mild release of pressure compared to sudden release may suppress the excitation of airway sensory C fibers and secondary neuroplasticity accompanied by cough. Also, these can decrease throat irritation and inflammation in turn [23]. In this circumstance, cough reflex may significantly be suppressed.

Postoperative airway complications in this study were mainly sore throat and hoarseness. They were caused by inflammation and stimulation of the airway due to the pressure exerted on the tracheal wall by the endotracheal [24], tracheal mucosal trauma, vocal cord hematoma, mucosal dehydration [25, 26], or laryngeal oedema [27–29]. As cuffs in group E were deflated passively, we found approximately 1 ml of gas left in several cuffs after extubation despite cuff pressures were zero. We estimated it was the cause of higher incidence of hoarseness in group E than in group C. To avoid pressure built-up when cuffs passing the vocal cords, we still attached pressure gauge when extubation.

Hans-Joachim P reported when the cuffs deflated, positive airway pressure cannot be maintained until the moment of extubation, and effective lung inflation prior to extubation cannot be performed. This hinders the generation of cough, and impair effective removal of secretions from glottic and subglottic areas during extubation. So, the report recommended to remove endotracheal tube while the lungs are inflated by positive pressure with endotracheal tube cuffs inflated, it generates an effective cough to expel secretions and blood from the glottic and subglottic areas during tracheal extubation [30, 31]. On this basis, we estimated in these study, sore throat may associated with the injury and stimulation on the trachea when intubation and cuff stimulation during operation, other airway complications can related to secretions descending along the trachea when extubation. And that’s the reason why continuous and slow deflation does not decrease the incidence of total postoperative airway complications, which was contrary to our expectations. Because we predicted that stimulation intensity on trachea when extubation was reduced and cough reflex was suppressed, the incidence of airway complications such as postoperative sore throat may decrease as many complications was likely mediated by mucosal trauma and inflammation, which was also associated with cough reflex [16]. All of these airway complications were cured within 7 days after surgery without specific treatment.

Should we decrease the incidence of cough reflex when extubation? Although cough is a protective mechanism, there are circumstances patients are vulnerable and susceptible to coughing. For example, cough may trigger an abrupt increase in intracranial, intracavity, or intraocular pressure, and these can lead to hypertension, tachycardia, arrhythmia, and cardiovascular collapse. Also, they may induce bronchospasm, laryngospasm, and other airway complications especially in asthma patients, children with upper respiratory tract infections (URIs). In these patients, prevention of cough reflex is warranted. However, before medication prevention for the suppression of cough reflex, the benefit and potential harm must be carefully weighed. For instance, patients undergoing cardiac surgery, with arrhythmia or interventional cardiac procedures should avoid lidocaine [16]. Therefore, the method of deflation a tracheal tube cuff to avoid cough reflex provides more clinical options. We hold the opinion that we should selecte an appropriate method according to the patient’s conditions and type of surgeries to avoid different postoperative complications.

This trial has some advantages. No study has investigated the effect of extubation using a cuff pressure gauge, and we designed a group that used a 10-ml syringe at 1 ml s^−1^ to extubate, which is more convenient in clinical practice. The trial, on the other hand, has flaws [32]. First, this was a single-centre study, and perhaps a multicentre study would have been better to further test our hypothesis. Second, as we can not measure the cuff volumes by deflating with a cuff pressure gauge, we did not account for it when extubation. As the cuff is inflated with the least amount of ordinary temperature air to create a seal during positive ventilation and minimize the pressure transmitted to the tracheal mucosa, 5-8 ml ordinary temperature air was recommend to inflation [33], we designed deflating cuffs via a cuff pressure gauge in 5 s, which expected to be comparable to the speed of deflating with a 10-ml syringe at 1 ml s^−1^. Third, the operation duration was within 3 h, and the effect of continuous and slow deflation in longer procedures is unknown.

In conclusion, this study demonstrated that in patients with normal BMI undergoing operations within 3 h, deflating the tracheal tube cuff continuously and slowly until the pressure was zero in 5 s using a cuff pressure gauge or using a 10-ml syringe at 1 ml s^−1^ can both reduce the incidence and severity of cough reflex during extubation, but deflating with a cuff pressure guage can increase the incidence of postoperative hoarseness.

## Data Availability

The datasets used and/or analyzed during the current study are available from the corresponding author on reasonable request.

## Abbreviations

BIS: Bispectral index
TOF: Train of four ratio
MAP: Mean artery pressure
HR: Heart rate
LSD: Least signficant difference
URI: Upper respiratory tract infection
